# Diagnostic Value of Plasma MicroRNAs for Lung Cancer Using Support Vector Machine Model

**DOI:** 10.7150/jca.30528

**Published:** 2019-08-28

**Authors:** Wei Wang, Mingcui Ding, Xiaoran Duan, Xiaolei Feng, Pengpeng Wang, Qingfeng Jiang, Zhe Cheng, Wenjuan Zhang, Songcheng Yu, Wu Yao, Liuxin Cui, Yongjun Wu, Feifei Feng, Yongli Yang

**Affiliations:** 1 Department of Occupational Health and Occupational Disease, College of Public Health, Zhengzhou University, Zhengzhou, China; 2 The Key Laboratory of Nanomedicine and Health Inspection of Zhengzhou, Zhengzhou, China; 3 Department of Environmental Health, College of Public Health, Zhengzhou University, Zhengzhou, China; 4 Department of Thoracic Surgery, the Affiliated Cancer Hospital of Zhengzhou University (Henan Cancer Hospital), Zhengzhou, China; 5 Department of Respiratory Medicine, The First Affiliated Hospital of Zhengzhou University, Zhengzhou, China; 6 Department of Public Health and Preventive Medicine, School of Medicine, Jinan University, Guangzhou, China; 7 Department of Sanitary Chemistry, College of Public Health, Zhengzhou University, Zhengzhou, China; 8 Department of Health Toxicology, College of Public Health, Zhengzhou University, Zhengzhou, China; 9 Department of Epidemiology and Biostatistics, College of Public Health, Zhengzhou University, Zhengzhou, China

**Keywords:** Lung cancer, Plasma miRNAs, Support vector machine, Diagnosis

## Abstract

**Aim**: Small single-stranded non-coding RNAs (miRNAs) play an important role in carcinogenesis through degrading target mRNAs. However, the diagnostic value of miRNAs was not explored in lung cancers. In this study, a support-vector-machine (SVM) model for diagnosis of lung cancer was established based on plasma miRNAs biomarkers, clinical symptoms and epidemiology material.

**Methods**: The expressions of plasma miRNA were examined with SYBR Green-based quantitative real-time PCR.

**Results**: We identified that the expressions of 10 plasma miRNAs (miR-21, miR-20a, miR-210, miR-145, miR-126, miR-223, miR-197, miR-30a, miR-30d, miR-25), smoking status, fever, cough, chest pain or tightness, bloody phlegm, haemoptysis, were significantly different between lung cancer and control groups (*P*<0.05). The accuracies of the combined SVM, miRNAs SVM, symptom SVM, combined Fisher, miRNAs Fisher and symptom Fisher were 96.34%, 80.49%, 84.15%, 84.15%, 75.61%, and 80.49%, respectively; AUC of these six model were 0.976, 0.841, 0.838, 0.865, 0.750, and 0.801, respectively. The accuracy and AUC of combined SVM were higher than the other 5 models (*P*<0.05).

**Conclusions**: Our findings indicate that SVM model based on plasma miRNAs biomarkers may serve as a novel, accurate, noninvasive method for auxiliary diagnosis of lung cancer.

## Introduction

Lung cancer is currently the number one cause of morbidity and mortality worldwide 1, which has been classified into small cell lung cancer (SCLC) and non-small-cell lung cancer (NSCLC). NSCLC could account for 85% of all lung cancers to become the main subgroup of lung cancer 2. NSCLCs can be further divided into adenocarcinoma (AC), squamous cell carcinoma (SCC) and large cell carcinoma (LCLC) three major histological subtypes 3. Yet the NSCLC patients at an early stage with no obvious clinical symptoms, and lack of sensitive biomarkers and effective tools for early diagnosis, therefore more than 75% of NSCLC patients are still diagnosed at advanced stages with distant metastases 4. Although novel therapies are improving the survival of lung cancer patients, 5-year survival rate was still less than 15% for advanced NSCLCs. However, the 5-year survival rate was up to 80% for the initial stage NSCLCs 5. Therefore, early diagnosis and early screening of lung cancer are particularly important.

Lung cancer is diagnosed by means of histological examination, diagnostic imaging, low-dose spiral computed tomography (LDSCT) and positron emission tomography (PET). Although these techniques have been improved, they still have some limitations and the five-year death rate of lung cancer remains low 6. For example, histological examination is the golden standard for diagnosis of lung cancer, but it is not suitable for early screening of lung cancer because of its traumatic and highly technical requirements. Diagnostic imaging such as Chest X Ray (CxR) and Computed Tomography (CT) have been used for diagnosing NSCLC at an early stage, however, there is a certain radiation hazard and limited role in reducing lung cancer mortality 7. While lung cancer mortality is reduced by 20% in high-risk lung cancer patients through LDSCT method, the false-positive are as high as 90% 8. Although the sensitivity and specificity of PET method are up to 90%, there is still a 10% false-positive rate and the cost is expensive 9. Therefore, new biomarkers and therapeutic strategies urgently need to be developed for better management of lung cancer.

miRNAs are small single-stranded non-coding RNAs that play vital regulatory roles by targeting mRNAs for degradation or translational repression. It acts as key regulators of cell proliferation, differentiation, apoptosis and other biological processes 10. A line of studies suggest that miRNAs are involved in human diseases and cancers. miRNAs expression is associated with lung cancer has been identified in varieties of normal and cancer tissues 11, 12. Moreover, it has been demonstrated that plasma miRNAs regulate numerous target genes and play a critical role in lung carcinogenesis, which indicates that miRNAs might be a potential diagnostic tool for lung cancer 13. Published studies 14-16 have shown that 11 plasma miRNAs (miR-16, miR-21, miR-20a, miR-210, miR-145, miR-126, miR-223, miR-197, miR-30a, miR-30d, miR-25) in lung cancer patients are abnormal expressions. These results suggest that combined with several miRNAs can improve the sensitivity and specificity for the early diagnosis of lung cancer.

Data mining(DM), also called Knowledge Discovery in Database(KDD), is extracting potentially useful information and knowledge of the process from abundant, incomplete, noisy, fuzzy and random practical application data 17. DM has a unique advantage in solving multi-parameter problems. Classification is part of the important functions of data mining, which is often closely related to disease diagnosis. At present, data mining is used primarily in the field of auxiliary diagnosis of diseases 18. DM techniques include SVM, artificial neural networks (ANN), decision tree (DT), genetic algorithms and so on. SVM is a pattern recognition method based on statistical learning theory (SLT) and structural risk minimization, which has several advantages such as prominent generalization ability and non-linear processing capacity and high-dimensional processing capacity in many areas 19.

Based on the previous research 20, this study explored the significance of the SVM model by using data of plasma miRNAs biomarkers for the auxiliary diagnosis of lung cancer.

## Materials and methods

### Study population

The lung cancer patient group consisted of 148 cases (age rank 29-87 years) with primary lung cancer from the First Affiliated Hospital of Zhengzhou University, Henan Cancer Hospital and Henan Provincial Chest Hospital, from Jun. 2016 to Feb. 2017. Patients were selected on the basis of the following inclusion criteria: (1) patients had a pathological diagnostic primary lung cancer that met histological or cytological criteria; (2) without undergoing surgical resection, chemotherapy, or radiotherapy; (3) without previous other organ tumors; (4) good compliance and availability of outcome data. Patients were excluded with major organ function failure, pregnant, or lactating. Pathologic diagnosis was based on WHO criteria. Lung cancer staging for each patient was performed according to the AJCC Cancer Staging Manual, 7th edition.

Controls come from a company who take physical examinations in Qixian Center for Disease Control and Prevention. The controls were excluded according to the following criteria: (1) without malignant tumors of the lung or other organs; (2) without major organ function failure; (3) without pregnant or lactating; (4) good compliance and availability of outcome data. A total of 148 gender- and age-frequency matched (±3 years) were enrolled in this study. The permission was got from each participant. A questionnaire that included the information of epidemiology was completed for each participant by trained interviewers. Smokers are defined as people who have smoked for six months or more in their lifetime according to the criteria of WHO. The alcohol-drinkers are defined as drinking alcohol at least once a week and the consumption of pure alcohol is above 20 g.

### Main instruments and reagents

The instruments and reagents used in the study included a Labcycler PCR amplifier (SensoQuest Company, China), a 7500 Fast Real-time PCR system (ABI, America), primers (Sangon Biotech), miRcute miRNA extraction and separation kit(Tiangen, Beijing), MiRcute enhanced miRNA fluorescence quantitative detection kit(Tiangen, Beijing) and ChemiDoc MP gel imaging analyzer(Bio-RAD, America).

### Statistical analysis and model evaluation

The Ct values of the samples were calculated with the software for real-time PCR instrument. The comparison of multiple of the expression of miRNA in the lung cancer patients to the normal controls was calculated using the formula of 2^-ΔΔCt^ (ΔCt= Ct_miR_ - Ct_external reference_; ΔΔCt=ΔCt_miR_ -ΔCt_average normal controls_).

The data was analyzed using SPSS 21.0 software. SPSS Clementine 21.0 software was used for data mining. The analysis of the quantitative data was analyzed with independent sample t-test or Mann-Whitney U. Each contingency table was tested by Chi-Square test. Binary logistic regression was conducted to analyse the influencing factors of lung cancer. The significance level was set at 0.05.

This study assessed sensitivity, specificity, accuracy positive predictive value (PPV), negative predictive value (NPV), and area under the ROC curve (AUC) to estimate the models.

### Establishment of models

#### Data preprocessing

Data transformation: The relative expression of 11 miRNAs did not follow a normal distribution, so normal transformation was needed. The expression of 11 miRNAs was normalized based on 10 common logarithm transformations.

Groups of training set and validation set: Based on the random sampling function of the partition node, according to a ratio of 3:1, the normalized data of each group were separated randomly into a training set (114 controls, 100 cancer cases) and a validation set (34 controls, 48 cancer cases). The training set was utilized to develop the model, while the validation set was used to verify the model.

#### Model derivation

The Data node is the source of data for the study; the variables are documented using Type node; the samples were randomly divided into the training set and validation set according to the proportion of 3:1 using Partition node; Random number seed is 1111111.

The Fisher and SVM models were developed using the training set, then the samples of the validation set were used to validate the quality of the models. The Combined model (16 items) with 16 input variables of smoking status, fever, cough, chest pain or tightness, bloody phlegm, hemoptysis and expressions of 10 plasma miRNAs (miR-21, miR-20a, miR-210, miR-145, miR-126, miR-223, miR-197, miR-30a, miR-30d, miR-25); the miRNAs model (10 items) with 10 input variables of plasma miRNAs (miR-21, miR-20a, miR-210, miR-145, miR-126, miR-223, miR-197, miR-30a, miR-30d, miR-25); the symptom model (6 items) with 6 input variables of fever, cough, chest pain or tightness, bloody phlegm, hemoptysis.

#### Fisher discrimination model

Fisher discrimination is a widely used classification model in traditional statistical methods. The basic idea: Projection before discriminant analysis, Projection is the core of the Fisher discrimination analysis. After repeating training, the Fisher discrimination parameter settings were: Use partitioned data: no; method: Enter; Mode: Expert; Prior probabilities: All groups equal; Use covariance matrix: Within-groups.

#### SVM model

The basic principle is to transform the input space into a high dimensional space by using the nonlinear transformation defined by the inner product function, and to find the optimal linear classification surface.

After repeating training, the SVM parameter settings were: Use partitioned data: no; Mode: Expert; Kernel type: Polynomial; Gamma: 1; Stopping criteria: 1.0E-3.

## Results

### Demographic characteristics of lung cancer patients and controls

The 148 lung cancer patients (mean age 60.97 ± 10.83 years) and 148 controls (mean age 60.14 ± 9.66 years) were enrolled. The age distribution of subjects was in normal distribution, so the age group was divided into two groups according to mean age (60 years). All the subjects were divided into four groups (Never smoking; Light smoking: <10 cigarettes/day; Moderate smoking: 10~20 cigarettes/day; Heavy smoke>20 cigarettes/day) according to the smoking status. As shown in Table 1, the average age, sex and alcohol were no significant differences between the two groups (*P*>0.05). However, the frequency of smoking, fever, cough, chest pain or tightness, bloody phlegm and hemoptysis were significantly higher in the cancer group than that in control group (*P*<0.001).

### Clinical pathologic characteristics of lung cancer patients

The clinical and pathological characteristics of lung cancer patients collected in this study are shown in Table 2. The lung cancer group was consisted of 36 SCC cases, 18 SCLC cases, 66 AC cases, 2 LCLC cases, and 26 other histological type cases; 33 cases of clinical stage Ⅰ and Ⅱ, 101 cases of clinical stage Ⅲ and Ⅳ.

### Comparison of the expressions of 11 plasma miRNAs between the two groups

As seen in Table 3, expressions of 10 plasma miRNAs (miR-21, miR-20a, miR-210, miR-145, miR-126, miR-223, miR-197, miR-30a, miR-30d, miR-25) were all significantly up-regulated in lung cancer patients than controls (*P*<0.05). However, the expression of miR-16 was no significant difference between lung cancer patients and controls (*P*>0.05).

### Data mining

The data of the Fisher and SVM model based on the smoking status, fever, cough, chest pain or tightness, bloody phlegm, haemoptysis and expressions of 10 plasma miRNAs (miR-21, miR-20a, miR-210, miR-145, miR-126, miR-223, miR-197, miR-30a, miR-30d, and miR-25) are presented in Table 4. In the training set, the accuracies of combined Fisher, miRNAs Fisher, symptom Fisher, combined SVM, miRNAs SVM, and symptom SVM model were 87.38%, 74.77%, 79.91%, 98.13%, 85.51%, and 83.64%, respectively. The accuracies in the validation set were 84.15%, 75.61%, 80.49%, 96.34%, 80.49%, and 84.15%, respectively.

### The evaluation of models

The results of the evaluation indexes of the 6 models were presented in Table 5. Sensitivity of combined SVM model reached 97.90%, and the specificity was 94.10%. PPV and NPV were likewise highest. Meanwhile, AUC was greater than 0.9. On the other hand, AUC of the miRNAs Fisher and symptom Fisher models were slightly smaller than the other models.

The results of the AUC of the 6 models were shown in Table 6. The AUC of combined SVM model was superior to the other 5 models, and the difference was statistically significant (*P*<0.05); The AUC of combined Fisher model was higher than miRNAs Fisher model and symptom Fisher model (*P*<0.05). There were no statistical differences in AUC among the other 3 models (*P*>0.05).

## Discussion

Early diagnosis and effective treatment of lung cancer is the key to improve the survival rate of patients. Therefore, early and non-invasive biomarkers for lung cancer diagnosis have been the most popular research areas. It has been shown that circulating miRNAs are stable under the actual experimental conditions and that abnormal expression of cancer-related miRNAs may be earlier than the clinical symptoms, therefore, circulating miRNAs may be used as tumor biomarkers 21. A large body of studies have suggested that a series of circulating miRNAs have the potential as diagnostic tool in malignancies 22. It has been shown that four plasma miRNAs (miR-21, miR-126, miR-210, and miR-486) could differentiate NSCLC from controls with 86.22% sensitivity and 96.55% specificity, which also could to distinguish NSCLC with 73.33% sensitivity and 96.55% specificity in phase Ⅰ 23. In the plasma of NSCLC patients, one study identified 15 types of miRNAs associated with lung cancer tissues from the literature and found that the expression of miR-155, miR-197, and miR-182 were significant increase in phase Ⅰ 16. The sensitivity and specificity of diagnosis NSCLC patients were 81.33% and 86.76%, respectively.

In this study, we compared the expression of 11 plasma miRNAs in lung cancer patient to that in the controls. Single-factor analysis showed that the expressions of 10 plasma miRNAs (miR-21, miR-20a, miR-210, miR-145, miR-126, miR-223, miR-197, miR-30a, miR-30d, miR-25) in lung cancer group were statistically significant higher than the controls; Multiple factor analysis revealed that elevated plasma miR-20a levels and miR-223 were risk factors for lung cancer.

The ten miRNAs explored in the present study have been evaluated as lung cancer markers in other studies. Potential mechanisms of the miRNAs on lung cancer were explored in previous study (Seen in Table 7), the main signaling pathways including PI3K/Akt/NF-Κb (miR-21, miR-223, miR-145, miR-126, and miR-30a) 24-28, STAT3 (miR-126, and miR-197) 27, 29, 30, estrogen (miR-21 and miR-210) 31, et al.

Transcription factors such as HMBOX1, DDX5, and ZBTB5, which were identified to be co-regulated by miR-20a and miR-15b, have implication on cancer progression 32. E2F1 and E2F8 belong to E2F transcription factor family that is essential for the regulation of cell cycle progression 33. miR-20a has been shown to directly inhibit E2F1 transcription factor and highly express in NSCLC tissues 34. miR-223 suppressed the proliferation, migration and invasion of NSCLC cells through directly inhibition of E2F8 expression 35. Increased expression of miR-20a and miR-223 were found in lung cancer in our study, which could be used as a molecular biomarker in auxiliary diagnosis of lung cancer.

miR-25, acting as an oncogene or anti-oncogene located on chromosome 7p22.1, is involved in the development of multiple malignant tumors at the post-transcriptional level 36. The expression of miR-25 in NSCLC tissues was significantly higher than that in adjacent non-cancerous tissues, which plays a carcinogenic effect by regulating cell cycle element E2 and is associated with cancer cell for resistance, proliferation, metastasis, invasion, and so on 37. miR-21 is over expressed in a variety of diseases and negatively responsible for the regulation of tumor suppressor genes by participating in the proliferation, invasion, metastasis and vascular infiltration of tumor cells, thus promoting the development of tumor 38. miR-210 and hypoxia-inducible factor 1-alpha (HIF-1α) play a synergistic role in the proliferation, differentiation, apoptosis, angiogenesis, DNA damage repair and energy metabolism of the hypoxic cell 39. Study also demonstrated the high expression of miR-210 in advanced lung cancer 40. miR-197 is related to infiltration and metastasis of tumor cells and located on chromosome 1p13.3. Recent research bears out the miR-197/CKS1B/STAT3-mediated PD-L1 network in chemoresistant NSCLC, independent of immunosuppression signals 29. Another research found decreased expression of miR-197 induces p53-dependent lung cancer cell apoptosis, which may be oncogene 41. The results of the study are basically in line with these studies in which the increased expression of miR-25, miR-21, miR-197 and miR-210 in the plasma of lung cancer compared with controls. Taken together, these results reflect the reliability and stability of miR-25, miR-21, miR-197 and miR-210 as the lung cancer biomarkers.

The data of the expressions of miR-145 are not consistent in different studies. It has been shown that miR-145 is down-regulated in various malignancies including lung adenocarcinoma, which inhibited cell proliferation through targeting epidermal growth factor receptor (EGFR) and nucleoside diphosphate 1 (NUDT1) 42. However, Study found that the increased expression of miR-145 in the plasma lung cancer, which is consistent with our study 43. miR-126 may inhibit the proliferation of lung cancer cells and the expression of miR-126 was lower than normal tissue 44. miR-30a can inhibit the invasion and migration of lung cancer cells by directly inhibiting the expression of the snail 45. miR-30d could inhibit the cell proliferation and activity of NSCLC by directly regulating CCNE2 46. In this study, the relative expressions of miR-126, miR-30a and miR-30d in plasma lung cancer patients were greater than controls, and the data differed from the studies above.

Various data mining algorithms have been improved in recent years, such as cluster analysis, decision tree and rough set, ANN and genetic algorithm, SVM and fuzzy processing technology 47. Each method has advantages and limits as well as the applicable scope. Fisher discriminant analysis is one of the most widely used method in multivariate statistical pattern recognition, which requires the independent input variables without interaction effect and normal distribution and so on 48. Therefore, the analysis of the nonlinear system has a couple of limitations. In order to get the best generalization ability, based on the statistical learning theory of VC (Vapnik-Cher-Vonenkis) and structural risk minimization principle, SVM finds the best compromise between the complexity of the model and the ability to learn 49. SVM is a classical method in data mining. There are several advantages of SVM method. For example, structural risk minimization and good generalization ability, what is based on statistical learning theory 50. The second, SVM can achieve similar results with different kernel functions like ANN, which depends on the selected model 51. In general, SVM is the optimal solution in the existing information situation, which makes up for the deficiency of ANN in determining the reasonable structure and local optimal problem, and has a significant improvement in learning methods. This study deeply analyzed with more mature SVM algorithms employed in the medical field.

At present, some studies have mostly focused on one or several biomarkers using traditional analysis methods. One study explored serum miR-22, miR-125b, and miR-15b diagnosis compared with the current commonly used tumor marker CEA, which indicates that the diagnostic significance of these three serum miRNAs(AUC=0.725, 0.704, and 0.619) for NSCLC was higher than that of serum CEA (AUC=0.594) 52. Meanwhile, some studies focused on gene and other biomarkers using ANN or decision tree model and so on. The ANN and decision tree model of lung cancer based on the genetic polymorphism of *CYP1A*1, *GSTM*1, m*EH*, *XRCC*1, the length of telomere, and the methylations of p16 and RASSF1A gene, the results showed that the accuracy for ANN and decision tree model validation sets was 89.62% and 93.00% 53. The accuracy and sensitivity were also improved by the above methods. In this study, the SVM model and Fisher model were established based on miRNAs tumor biomarkers and clinical symptom characteristics for the first time.

We established the Fisher model with 10 miRNA and 6 symptom for lung cancer diagnostic, and, the AUCs of three models are combined Fisher model (16 items) (0.865, 95%CI=0.821-0.902), miRNAs Fisher model (10 items) (0.750, 95%CI 0.697-0.798), and symptom Fisher model (6 items) (0.801, 95%CI 0.751-0.845), respectively. The accuracy for three model validation sets was 84.15%, 75.61%, and 80.49%, respectively. The combined Fisher model showed good ability to detect lung cancer, which is superior to the lung cancer diagnosis Fisher model (0.670, 95%CI 0.569-0.761) established with FHIT, RASSF1A, p16 promoter methylation, and relative telomere length in our prophase research 20. This may be due to the miRNAs biomarkers has better specificity compared with gene or other biomarkers. Our findings indicate that the changed expression levels could be used as potential biomarkers for diagnosis of lung cancer. Besides, probably because of the data pretreatment before model established. After the normal transformation, the expression levels of miRNAs are approximately normal distribution and without missing values.

miRNAs play a critical role in lung cancer carcinogenesis, which were studied widely as cancer biomarkers. Zhang et al 54 established screening method for early-stage NSCLC using four miRNAs (miR-145, miR-20a, miR-21, miR-223), and the AUC of the model was 0.897. To the best of our knowledge, there is no data mining model for lung cancer diagnosis based on miRNAs. SVM model were established for lung cancer diagnostic in our study, which combined 10 miRNAs and 6 symptoms, had a higher accuracy. The combined SVM model with miRNAs was superior in lung cancer diagnosis in this study compared to models with methylation and telomere biomarkers in our prophase research 20. The accuracy and AUC of combined SVM model in our study were also better than the results of other studies on gene and other biomarkers using ANN or SVM and so on. For example, one study explored eighteen genes (including TTN, RHOH, RPS20, TRBC2) for six cancer (including lung cancer) using SVM with accuracy of 75.10% 55.

As to the three models we established, the accuracy of models (10 miRNAs SVM, 6 symptom SVM model, and combined SVM) were 80.45%, 84.15%, and 96.34%, respectively; the AUC of models (10 miRNAs SVM, 6 symptom SVM model, and combined SVM) were 0.841, 0.818, and 0.976, respectively. The AUC and accuracy of combined SVM model were better than the miRNAs SVM and symptom SVM model. Overall, the SVM model based on miRNAs and clinical symptom characteristics has a higher accuracy rate and might be useful for early diagnosis of lung cancer, which also has excellent predictive power, such as all patients with stage Ⅰ and Ⅱ lung cancer in validation set were correctly predicted to be lung cancer.

This study showed that 10 plasma miRNAs expression levels were associated with lung cancer, which provides a theoretical possibility for further prospective studies or large-scale clinical trials. More importantly, the expression of the plasma miRNAs is very stable under different harsh conditions, which indicating that the plasma miRNAs has the potential to serve as biomarker for auxiliary diagnosis of lung cancer. Our findings indicate that SVM model based on plasma miRNAs biomarkers may serve as a novel, accurate, noninvasive method for auxiliary diagnosis of lung cancer. However, there are some limitations in this study. Firstly, the selection of 10 plasma miRNAs were based on published studies rather than miRNA array or bioinformatics method. More plasma miRNAs need to be analyzed to for using as specific biomarkers. Secondly, compare to single study, large sample and multicenter clinical trial studies will yield more reliable results. Moreover, there are still things for the further validation study need to be thought, including health policy, ethics, cost, et al.

## Conclusions

In summary, this study suggests that the 10 plasma miRNAs are associated with lung cancer, and the changed expression levels could be used as potential biomarkers for diagnosis of lung cancer. SVM model has the superior diagnostic value for auxiliary diagnosis of lung cancer based on miRNAs tumor biomarkers and clinical symptom characteristics.

## Figures and Tables

**Table 1:** Demographic characteristics of lung cancer patients and controls

Variable		Lung cancer (n=148)	Controls (n=148)	*χ^2^*/*t*	*P*
Age*		60.97±10.83	60.14±9.66	0.691	0.490
Age-grouped	≤60	67	68	0.014	0.907
	>60	81	80		
Gender	Male	98	99	0.821	0.365
	Female	50	49		
Fever	No	131	146	12.654	<0.001
	Yes	17	2		
Cough	No	57	130	77.387	<0.001
	Yes	91	18		
Chest pain or tightness	No	86	135	42.878	<0.001
	Yes	62	13		
Bloody phlegm	No	109	148	44.918	<0.001
	Yes	39	0		
Hemoptysis	No	134	148	14.695	<0.001
	Yes	14	0		
Weak	No	146	145	0.000	1.000
	Yes	2	3		
Alcohol	Never	134	129	0.853	0.356
	Yes	14	19		
Smoking status	Never	76	89	16.989	<0.001
	Light	10	26		
	Moderate	28	15		
	Heavy	34	18		

Note: The * indicates age according with normal distribution.

**Table 2:** Clinical and pathological characteristics of lung cancer patients and controls

Clinical and pathological characteristics		n	Percentage (%)
Histological type	SCLC	18	12.16
	SCC	36	24.32
	AC	66	44.59
	LCLC	2	11.35
	Others	26	17.57
TNM stage^*^	Ⅰ+Ⅱ	33	24.63
	Ⅲ+Ⅳ	101	75.37
lymphatic metastasis ^*^	No	21	17.80
	Yes	97	82.20
distant metastases^*^	No	83	70.34
	Yes	35	29.67

Note: The * indicates data is missing.

**Table 3:** The relative expression of 11 plasma miRNAs in lung cancer and controls

miRNAs	Lung cancer (*n*=148) M (*P*25, *P*75)	Control (*n*=148) M (*P*25, *P*75)	Z	*P*
miR-16	1.60(0.70,2.93)	1.39(0.66,2.51)	-1.184	0.236
miR-21	1.05(0.77,2.09)	0.68(0.53,0.90)	-6.017	<0.001
miR-20a	1.93(0.81,4.40)	0.80(0.42,1.51)	-6.264	<0.001
miR-210	1.10(0.53,3.09)	0.68(0.39,1.24)	-4.267	<0.001
miR-145	1.11(0.56,2.93)	0.70(0.44,1.07)	-4.242	<0.001
miR-126	1.64(0.71,2.83)	0.77(0.32,1.58)	-5.096	<0.001
miR-223	2.26(1.26,5.55)	0.76(0.41,1.36)	-8.952	<0.001
miR-197	1.13(0.59,2.29)	0.59(0.41,1.25)	-5.008	<0.001
miR-30a	0.82(0.51,2.81)	0.66(0.37,1.75)	-2.908	<0.001
miR-30d	1.37(0.78,3.55)	0.69(0.48,1.24)	-6.409	<0.001
miR-25	1.36(0.77,3.27)	0.80(0.34,1.73)	-4.925	<0.001

**Table 4:** Effect of data mining on distinguish lung cancer

Model		Training set(n=214)		Validation set(n=82)	
		Cancer cases	Controls	Cancer cases	Controls
Combined Fisher model	Cancer cases	79	21	41	7
	Controls	6	108	6	28
	Total	85	129	47	35
	Accuracy	87.38%		84.15%	
miRNAs Fisher model	Cancer cases	70	30	38	10
	Controls	24	90	10	24
	Total	94	120	48	34
	Accuracy	74.77%		75.61%	
Symptom Fisher model	Cancer cases	71	29	38	10
	Controls	14	100	6	28
	Total	85	129	44	38
	Accuracy	79.91%		80.49%	
Combined SVM model	Cancer cases	99	1	47	1
	Controls	3	111	2	32
	Total	102	112	49	33
	Accuracy	98.13%		96.34%	
miRNAs SVM model	Cancer cases	83	17	38	10
	Controls	14	100	6	28
	Total	97	117	44	38
	Accuracy	85.51%		80.49%	
Symptom SVM model	Cancer cases	81	19	42	6
	Controls	16	98	7	27
	Total	97	117	49	33
	Accuracy	83.64%		84.15%	

**Table 5:** Comparison results in the validation set by SVM and Fisher models

Model		Sensitivity (%)	Specificity (%)	Accuracy (%)	PPV (%)	NPV (%)	AUC (95% CI)
Fisher	Combined	0.854	0.824	0.842	0.872	0.800	0.865(0.821,0.902)
	miRNAs	0.792	0.706	0.756	0.792	0.706	0.750(0.697,0.798)
	Symptom	0.792	0.824	0.805	0.864	0.737	0.801(0.751,0.845)
SVM	Combined	0.979	0.941	0.963	0.959	0.970	0.976(0.952,0.990)
	miRNAs	0.792	0.824	0.805	0.864	0.737	0.841(0.795,0.881)
	Symptom	0.875	0.794	0.842	0.857	0.818	0.838(0.791,0.878)

**Table 6:** Comparison of results in validation set by SVM and Fisher discriminant analysis

Comparison of models	Z	*P*
Combined SVM model *vs* Combined Fisher model	5.474	<0.0001
Combined SVM model *vs* miRNAs SVM model	6.445	<0.0001
Combined SVM model *vs* Symptom SVM model	6.363	<0.0001
miRNAs SVM model *vs* Symptom SVM model	0.105	0.9168
miRNAs SVM model *vs* miRNAs Fisher model	4.032	0.0001
Symptom SVM model *vs* Symptom Fisher model	2.256	0.0241
Combined Fisher model *vs* miRNAs Fisher model	4.179	<0.0001
Combined Fisher model *vs* Symptom Fisher model	3.167	0.0015
miRNAs Fisher model *vs* Symptom Fisher model	1.454	0.1459

**Table 7:** Potential mechanisms of the miRNAs on lung cancer

miRNAs	Pathways
miR-20a	angiogenesis, TGF-β pathway, platelet-derived growth factor pathway, and oxidative stress response^32^
miR-21	PI3K/AKT/NF-κB signaling pathway, and estrogen signaling pathway^24^; autophagy-related AMPK/ULK1 signaling pathway^56^
miR-210	estrogen signaling pathway^31^
miR-223	Notch/miR-223/FBXW7 pathway^57^; NF-κB signaling pathway^58^; IGF-1R/Akt/S6 signaling pathway^25^; IGF-1R/PI3K/AKT signaling pathway^59^
miR-25	ERK signaling pathway^60^; cell cycle regulation^61^
miR-145	ERβ/MALAT1/miR145-5p/NEDD9 signaling pathway^62^; EGFR/PI3K/AKT signaling pathway^26^; JNK signaling pathway^63^; mTOR signaling pathway^64^
miR-126	STAT3 signal pathway^30^; PI3K/AKT/Snail signal pathway^27^
miR-30a	PI3K/AKT signaling pathway^28^
miR-197	miR-197/CKS1B/STAT3-mediated PD-L1 network^29^

## Abbreviations

Adenocarcinoma: AC
Artificial neural networks: ANN
Area under the ROC curve: AUC
Data mining: DM
decision tree: DT
Knowledge Discovery in Database: KDD
Large cell carcinoma: LCLC
Negative predictive value: NPV
Positron emission tomography: PET
Positive predictive value: PPV
Received operating characteristic: ROC
Small single-stranded non-coding RNAs: miRNAs
Support vector machine: SVM
Small cell lung cancer: SCLC
Non-small-cell lung cancer: NSCLC
Squamous cell carcinoma: SCC
